# Adalimumab in the Treatment of Recalcitrant Livedoid Vasculopathy

**DOI:** 10.7759/cureus.50053

**Published:** 2023-12-06

**Authors:** Drishti M Bhatt, Sangeeta Bhamburkar, Bhushan Madke, Shivani D Jangid, Arshiya Khan

**Affiliations:** 1 Dermatology, Venereology and Leprosy, Jawaharlal Nehru Medical College, Datta Meghe Institute of Higher Education and Research, Wardha, IND; 2 Dermatology, Venereology and Leprosy, Bhamburkar Hospital and Skin Clinic, Akola, IND

**Keywords:** occluding vasculopathy, tnf-α inhibitor, adalimumab, non-healing ulcer, atrophie blanche, tnf-α, livedoid vasculopathy

## Abstract

Livedoid vasculopathy is a rare condition affecting the cutaneous vasculature. Patients typically develop bilateral lower limb ulcers that tend to recur and do not heal. Edema, discomfort, and itching are linked to ulcers. The patient’s quality of life is negatively impacted by this. *Atrophie blanche*, a stellate, porcelain-white scar, is typically left behind once these ulcers heal. Livedoid vasculitis, livedo reticularis with ulcerations, *atrophie blanche*, segmental hyalinizing vasculitis, and painful purpuric ulcers with a reticular pattern on the lower limbs are some of the terminologies used to describe livedoid vasculopathy. This condition has been treated using various techniques, including intravenous immunoglobulins, steroids, anticoagulants, antibiotics, immunosuppressive drugs, and antiplatelets. Here, we report the case of a 24-year-old male who presented with a red-colored, painful, and itchy lesion over his right calf for one year. He had recurring lesions over his right foot for the past 18 years. He had received multiple treatment courses over 18 years but had no relief. He was treated with eight doses of adalimumab injection (40 mg/0.8 mL) administered subcutaneously at an interval of 15 days. He had near-complete healing of the ulcer and complete remission of symptoms.

## Introduction

Livedoid vasculopathy (LV) is a rare entity of hyalinizing, chronic vascular disease with cutaneous symptoms. These patients have a significantly lower quality of life as they constantly experience pain and hyperesthesia in the vicinity of the affected area [1]. The pathophysiology of LV may involve plasminogen activator inhibitor (PAI)-1 as an etiological factor, according to a recent review by Huang et al. [1]. The endogenous fibrinolytic system in blood coagulation is inhibited by PAI-1, which has recently gained interest because of its possible involvement in the pathophysiology and mechanism of LV. According to reports, inflammatory cytokines, such as tumor necrosis factor-alpha (TNF-α), are responsible for regulating the expression of the genes that affect endothelial cell function and cause PAI-1 synthesis. Thus, TNF-α might play an indirect role in the development of LV [1]. It is an occlusive condition that occurs recurrently in the dermal vascular microcirculation. The presentation of symptoms includes livedo racemosa, a painful ulceration located more commonly in the distal parts of the lower limb extremities. This lesion is followed by healing as porcelain-white, atrophic scars known as atrophie blanche. Considerable pain results from ischemia caused by occlusive vasculopathy of the vessels present in the dermis [2]. During the evolution of the LV lesion, erythematous papules with purpura, plaques, and papules are formed. These lesions are painful on palpation and sometimes may be itchy on presentation. Some lesions can evolve into hemorrhagic vesicles. Punctate telangiectasias in the periphery of the ulcer and slough can appear in some areas that are about to ulcerate [3].

Deficiencies of protein C and protein S, factor V Leiden mutation, antithrombin III, prothrombin gene mutation, and hyperhomocysteinemia are among the significant procoagulant factors found to be responsible for the pathogenesis of LV. Livedoid vasculitis, segmental hyalinizing vasculitis, atrophie blanche, livedo reticularis with ulcerations, and painful purpuric ulcers with a reticular pattern on the lower limbs are some of the terminologies used to describe LV. Defects in endothelial cell plasminogen activation, platelet dysfunction, or increased fibrin production can lead to thrombotic consequences. Diffusion barrier occurs by fibrin deposition in pericapillary development and thrombus formation. This reduces the oxygen flow to the tissue, which causes an ischemic infarction. Poor tissue perfusion makes wound healing difficult. Sluggish circulation increases the risk of infection because leucocytes are unable to kill microorganisms effectively. Tissue perfusion is further threatened due to a vicious cycle, including tissue lysis, edema, and thrombosis [4]. Adalimumab is a high-affinity recombinant monoclonal antibody against TNF-α that is entirely of human origin. It does not interact with or bind to interleukins or lymphotoxin, among other cytokines [5].

## Case presentation

A 24-year-old male, a shopkeeper by occupation, with a height of 183 cm, weight of 102 kg, and body mass index of 30.4 kg/m^2^, presented with the chief complaint of a recurrent, non-healing ulcer over his right lower limb for the past year. The lesion was associated with pain, discharge, bleeding, and swelling. It was insidious in onset and gradually progressive. Upon further inquiry, the patient revealed that he had developed multiple similar painful ulcers over his right lower limb for the past 18 years. The lesions would heal with scarring. The patient had been a tobacco chewer for 12 years and had been consuming alcohol regularly for 12 years. The patient received multiple courses of oral and topical steroids, antibiotics, antifungals, oral fibrinolytics, vasodilators, and anti-inflammatory agents over 18 years but had partial relief in symptoms. He would experience temporary relief for the treatment period, but the lesions would recur with the same symptoms and at the same site. Informed and written consent was taken from the patient for the adalimumab injection. Written consent was also obtained for utilizing the patient’s information, other than his identity, that would be concerned with the case for publication purposes. All possible outcomes and side effects of the injection were explained to the patient in detail before administering the injection.

**Figure 1:**
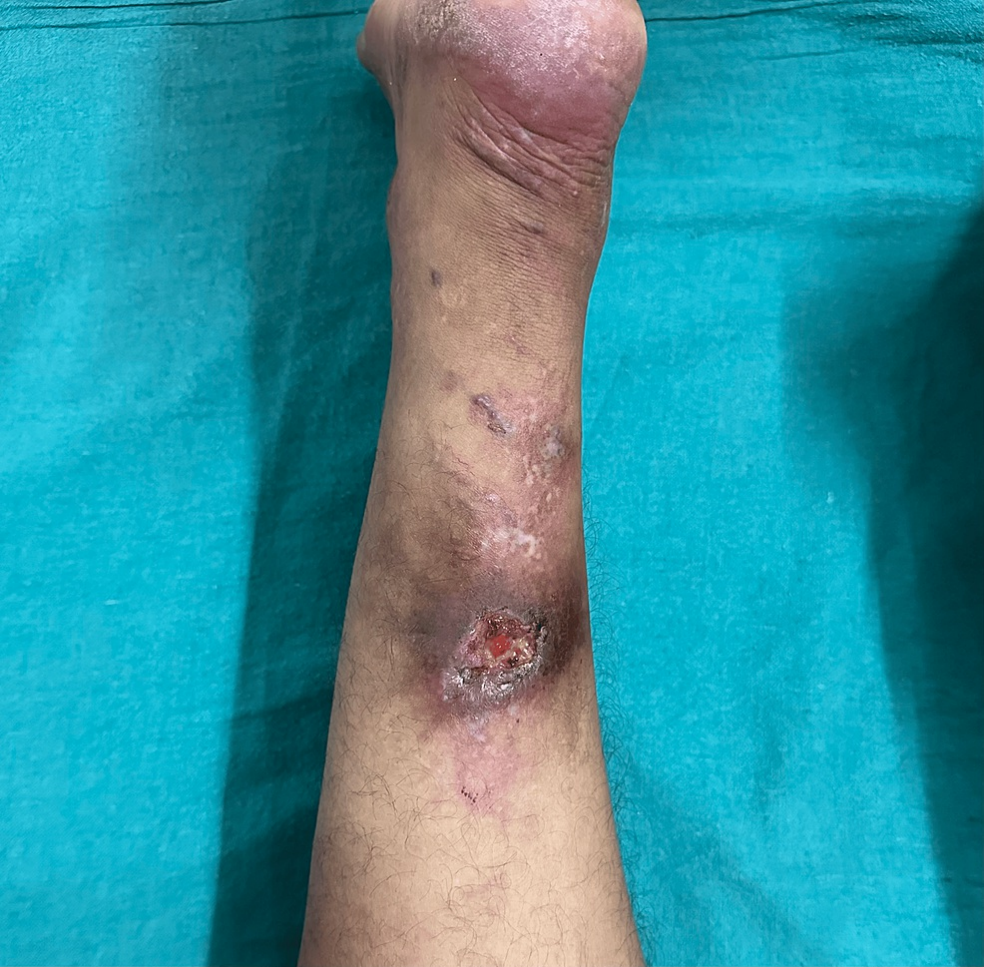
An ulcer measuring about 3 × 3 cm noted over the patient’s right lower calf with slough along with pus on the floor and sloping edges.

On examination, an ulcer measuring about 3 × 3 cm was present over his right lower calf with slough, pus, and sloping edges (Figure 1).

**Figure 2:**
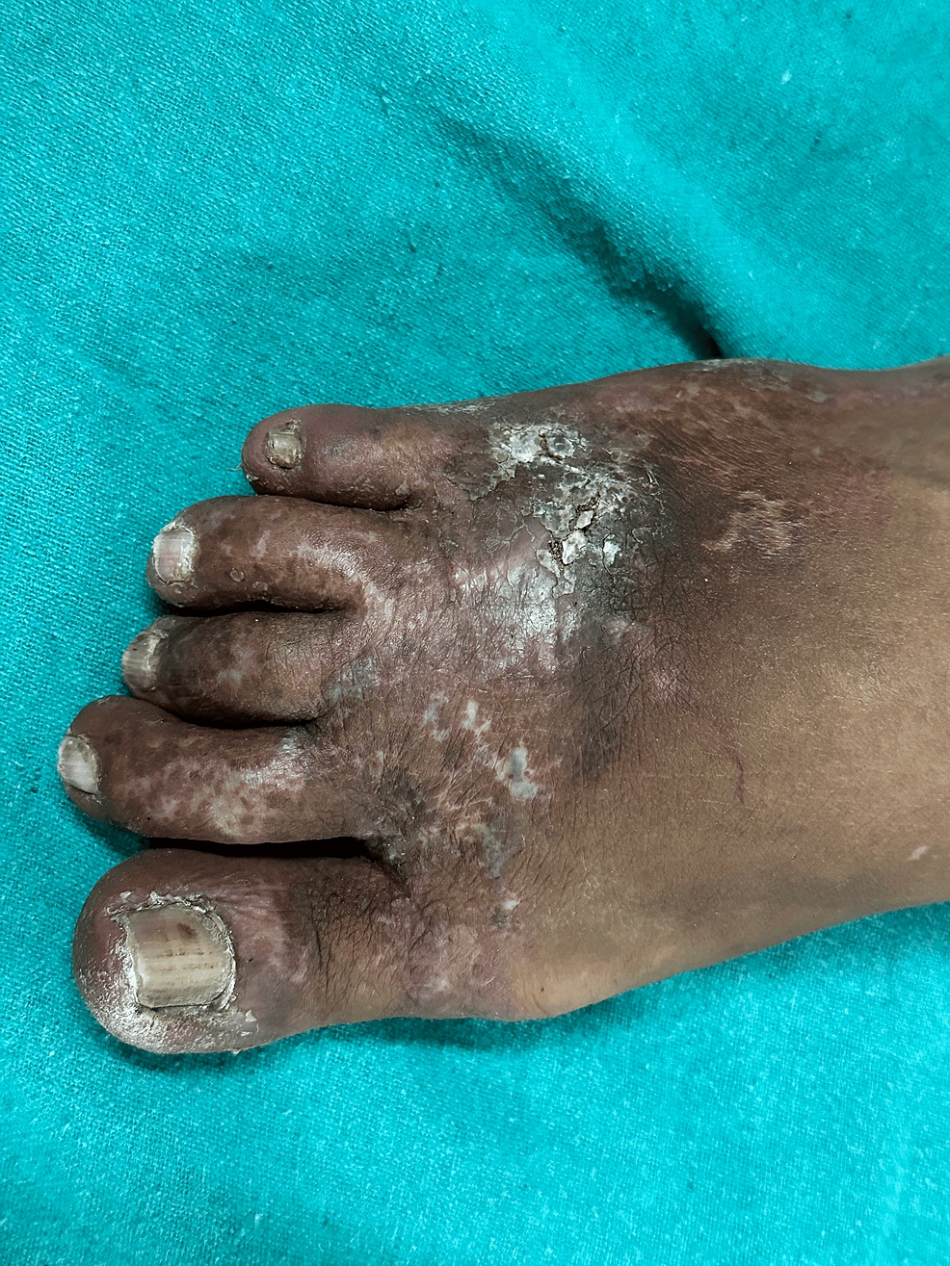
Erythema, eedema, and porcelain-white stellate scars with surrounding hyperpigmentation present over the dorsum of the right foot.

**Figure 3:**
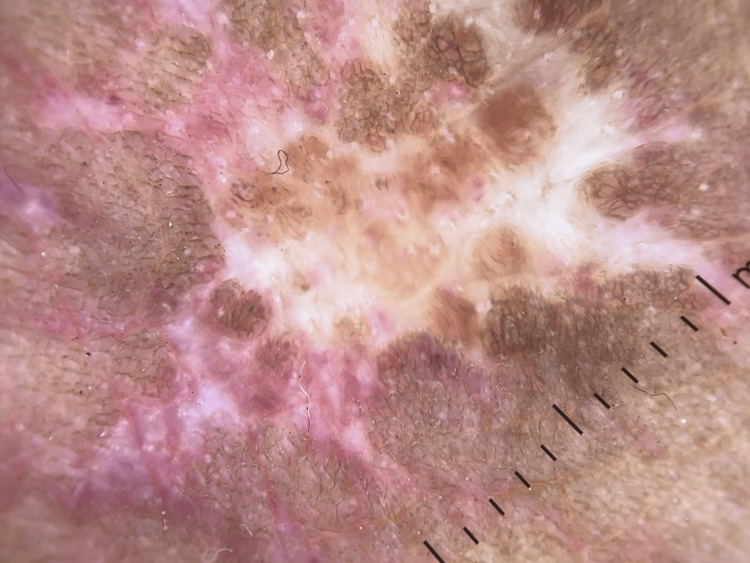
Dermatoscopy showing the presence of atrophie blanche and erythema over the dorsum of the patient’s right foot and over the area surrounding the ulcer.

Erythema was present on the surrounding skin. Edema and porcelain-white stellate scars with surrounding hyperpigmentation were present over the dorsum of the right foot (Figure 2). His complete hemogram and serum biochemistry for liver and renal function were within the normal range. Serology for retrovirus, hepatitis B virus, and hepatitis C virus were negative. Chest X-ray and Doppler study of the lower limb were normal. Dermatoscopy revealed the presence of atrophie blanche and erythema over the dorsum of his right foot and the area surrounding the ulcer (Figure 3).

**Figure 4:**
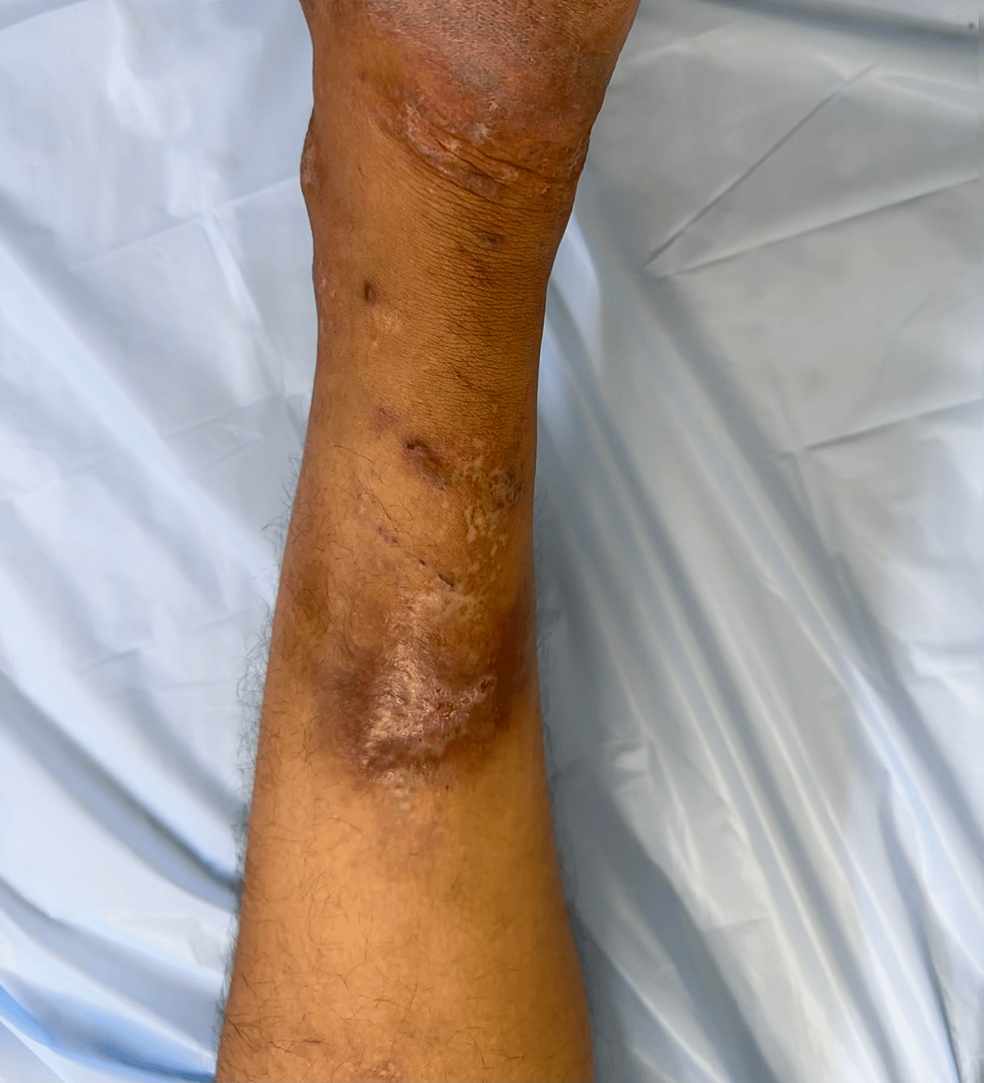
Completely healed ulcer with mild scarring and atrophie blanche after four months of treatment with adalimumab.

We prescribed fortnightly adalimumab injection (40 mg/0.8 mL) subcutaneously. After eight doses of adalimumab injection, the patient had near-complete remission of symptoms, and the ulcer completely healed with mild scarring and atrophie blanche (Figure 4). The patient did not report any side effects during the treatment and two months after completing the treatment course.

## Discussion

Micieli and Alavi [6] discovered that anticoagulants are the most frequently utilized monotherapy for LV, producing a positive result. The most often utilized treatment options, in order of utility, were anabolic steroids, intravenous immunoglobulins, and antiplatelets. Positive clinical results were linked to each of these treatments [6]. Multivitamins, systemic phototherapy, cyclosporin A, azathioprine, cyclophosphamide, and vasodilators such as nifedipine are further alternatives, as reported by Palanisamy et al., in the management of resistant cases of LV [7]. The patient had received multiple treatments in the past with no relief. We administered adalimumab injections to this patient with recalcitrant LV and achieved a positive outcome with the same.

Hairston et al. [8] found that, under the influence of medication or disease leading to a hypercoagulable state, patients with congenital thrombophilic disorders may be at an increased risk of thrombosis [8]. When treating patients with adalimumab, side effects, such as tuberculosis infection, deep fungal infections, and other uncommon diseases, must be considered. We did not encounter any such side effects during or after the treatment. Screening for these illnesses must be done. A complete panel of tests was conducted to rule out latent tuberculosis, bleeding disorders, or hepatitis infections, which could be reactivated if present. All tests were negative and we acquired fitness to administer adalimumab injection. Adalimumab should only be used cautiously in people with congestive heart failure or neurological diseases, which were absent in this patient. Patients need to be informed that they may have a higher risk of lymphoma, as pointed out by Ellis and Azmat [9]. The patient was counseled about all of the risks involved with the treatment and he provided his informed consent. Lu et al [10] investigated the safety of biosimilars of adalimumab and reported that every biosimilar switching trial showed no discernible difference in immunogenicity, safety, or efficacy when moving from adalimumab to a biosimilar [10].

## Conclusions

LV is a chronic condition of the cutaneous vessels. It presents with recurrent, painful ulcers and more frequently involves the lower limbs. Studies have shown the involvement of inflammatory cytokines such as TNF-α in the pathogenesis of LV. Multiple treatment modalities have been tried and proven successful for the treatment. Some cases might be resistant to these treatment options with only temporary relief, leading to a recurrence of symptoms. Adalimumab is a high-affinity recombinant monoclonal antibody with actions against TNF-α. In this case report, we presented the case of a patient with LV recalcitrant to multiple treatment modalities. We used adalimumab injection successfully in the treatment of resistant LV by administering eight doses, subcutaneously, over four months. There was complete remission of symptoms and healing of the ulcer, providing relief to the patient’s physical and psychological stress. He reported no side effects during or after the treatment. We presented a positive outcome of LV, with the subcutaneous administration of adalimumab injection over four months. This helps us conclude that adalimumab must be considered a valid option for the successful treatment of recalcitrant cases of LV with minimal side effects, as observed in this case.
